# DING Protein Inhibits Transcription of HIV-1 Gene through Suppression of Phosphorylation of NF-κB p65

**DOI:** 10.16966/2380-5536.175

**Published:** 2020-08-31

**Authors:** Nune Darbinian, Armine Darbinyan, Nana Merabova, Rebeccah Gomberg, Erik Chabriere, Malgorzata Simm, Michael E Selzer, Shohreh Amini

**Affiliations:** 1 Center for Neural Repair and Rehabilitation, Lewis Katz School of Medicine, Temple University, USA; 2 Department of Pathology, Yale University School of Medicine, USA; 3 Aix-Marseille Université, Institut Universitaire de France, IHU Mediterranée Infection, France; 4 University of Pikeville, Kentucky College of Osteopathic Medicine, USA; 5 Department of Biology, College of Science and Technology, Temple University, Philadelphia, USA

**Keywords:** DING, HIV-1, Tat, p65, p50, NF-κB

## Abstract

**Introduction:**

Novel plant DING proteins (full-length 38 kDa p38SJ, and 27 kDa p27SJ) exhibit phosphatase activity and modulate HIV-1 gene transcription. Previously, we demonstrated that DING regulates HIV-1 gene transcription by dephosphorylation and inactivation of CTD RNA polymerase II, the major elongating factor of HIV-1 Long Terminal Repeats (LTR). Because the transcription of HIV-1 is controlled by several viral and cellular factors, including p65/p50 subunits of NF-κB, we hypothesized that DING phosphatase can also affect the phosphorylation and activity of p65 NF-κB, in addition to C-terminal Domain (CTD) of RNA Polymerase II (RNAPII), to suppress HIV-1 gene transcription and inhibit HIV-1 infection.

**Methods:**

Here, we describe the inhibition of HIV-1 infection and the p65/p50 NF-κB phosphorylation by DING protein, analyzed by ELISA and northern-blot assays, western-blot assays, cell fractionation, and promoter-reporter assays in DING-expressing cells, using a pTet-on inducible system.

**Results:**

Results from HIV-1 infection assays demonstrate a strong inhibition of HIV-1 and HIV-LTR RNA expression by DING protein, determined by p24 ELISA and by northern blot assay. Results from the western blot assays and cell fractionation assays show that there is an increase in the level of hypo-phosphorylated form of p65 NF-κB in DING-expressing cells. Both fractions of p65/p50, nuclear or cytoplasmic, are affected by DING phosphatase, but more cytoplasmic accumulation of p65 NF-κB was found in the presence of DING, suggesting that subsequent activation and nuclear import of active NF-κB is affected by DING. The major portion of nuclear p65 was dephosphorylated in DING-expressing cells. The promoter-reporter assay demonstrated that DING-mediated dephosphorylation and dysregulation of NF-κB p65 lead to the suppression of its binding to HIV-1 LTR, and resulted in the inhibition of p65-mediated activation of LTR transcription. Mapping of the region within LTR that was affected by DING revealed that both, NF-κB and CTD RNA Polymerase II binding sites were important, and cooperativity of these cellular factors was diminished by DING. In addition, mapping of the region within DING-p38SJ that affected LTR transcription, revealed that phosphate-binding domain is essential for this inhibitory activity.

**Conclusion:**

We have demonstrated the effect of DING phosphatases on HIV-1 infection, phosphorylation of p65 NF-κB, and transcription of HIV-1 LTR. Our studies suggest that one possible mechanism by which DING can regulate the expression of HIV-1 LTR can be through dysregulation of the transcription factor NF-κB p65 by preventing its phosphorylation and translocation to the nucleus and binding to the HIV-1 LTR, an action that could contribute to the utility of DING p38SJ as an antiviral agent. Importantly, DING not only inhibits HIV-1 LTR gene transcription in the presence of increased p65 NF-κB, but also suppresses HIV-1 infection. DING protein improved inhibitory effects of the known anti-retroviral drugs, Tenofovir (TFV) and Emtricitabine (FTS) on HIV-1, since in the combination with these drugs; the suppression of HIV-1 by DNG was significantly higher when it was in combination with these drugs, compared to controls or cases without DING. Thus, our data support the use of neuroprotective DING proteins as novel therapeutic antiviral drugs that suppress HIV-1 LTR transcription by interfering with the function of NF-κB.

## Introduction

DING proteins (p27SJ and p38SJ) are plant phosphatases, isolated from *Hypericum perforatum* [1,2]. These DING proteins are implicated in neuroprotection [3,4], cell proliferation [5,6], and suppression of HIV-1 [1,2,7]. DING p27SJ and p38SJ belong to the DING family of proteins with conserved DINGG N-terminal region. DING proteins were isolated from animals, plants and prokaryotes, and some are characterized as phosphatases or phosphate-binding proteins [8]. The role of eukaryotic DING proteins on the transcription of HIV-1 LTR has been studied intensively [9-17]. Recently, it was determined that DING proteins from phylogenetically different species share similar sequence and structural homology and inhibit HIV-1 LTR transcription [18]. In previous studies, we showed that by inhibition of mitogen-activated protein kinase Erk1/2 phosphorylation, DING p27SJ suppresses phosphorylation of its substrate, Stat3 [5,6]. It was shown that NF-κB, which controls expression of pro-inflammatory genes, is activated during HIV-1 infection in humans [19-22]. HIV-1 Tat protein, in the presence of PMA, also can activate NF-κB through blocking of the binding of the inhibitor IκB-α repressor to the NF-κB complex [23]. Moreover, Tat protein can bind to the Interleukin-6 (IL-6) RNA, or interact with CAAT Enhancer-Binding Protein beta (C-EBPβ) transcription factor, to increase the expression of the IL-6 gene [24]. HIV Tat can interact with p65 NF-κB and increase its DNA-binding and transcriptional activity. Interaction of Tat with p65 and IκB-α can lead to NF-κB activation, and this mechanism can contribute to the dysregulation of the cellular response by HIV-1. The transcription of inflammatory response genes is regulated by NF-κB [25]. The NF-κB transcription factors include several proteins that contain Rel Homology Domain (RHD), such as RelA/p65, c-Rel, RelB, p50 and p52. RHD domain in 300-amino acid length is important for for homo- or hetero- dimerization, also for DNA-binding of NF-κB [26,27]. Dimerization is important for the transcriptional activity of NF-κB. Importantly, C-terminal transcriptional activation domains are present in p65 NF-κB [28]. When NF-κB associates with its inhibitors (IκB), this association affects its DNA-binding ability [29,30]. To activate NF-κB, IκB serine amino acids should be phosphorylated by the activated IκB Kinase (IKK). This causes the protein ubiquitination and degradation, and subsequent release and translocation of the functional NF-κB complex to the nucleus [31,32]. The most abundant NF-κB inhibitor is IκB-α [33-35]. I-κB is phosphorylated by IKK at serine residues, Ser32 and Ser36 [36,37]. The NF-κB activity can be further increased by PKA phosphorylation [38], or by PKCζ [39] and by IKKα [40]. NF-κB activation was shown in HIV-1-infected macrophages, monocytes, CD4 T lymphocytes and microglia [41]. Active NF-κB upregulates the expression of its responsive genes that include pro-inflammatory chemokines and cytokines [41]. Investigating the mechanisms of NF-κB or Tat inhibition could provide further insights into AIDS pathogenesis and treatment [42]. Viral HIV-1 Tat protein that interacts with the viral RNA and cellular factors can control viral replication [43-46]. To activate transcription, Tat usually binds to RNA stem-loop structures, including the HIV-1 transactivation-responsive element within the viral LTR. It alsointeracts with somecellular transcriptionfactors. Tat can be released from infected cells and bind to integrins to dysregulate the cell signaling [49], or to chemokine receptors [50,51]. Tat increases the p65 transcriptional activity and modulates other proteins that regulate NF-κB signaling. When Tat binds to IκB-α, it is translocated to the nucleus [52,53]. It is important to uncover mechanisms of the NF-κB suppression during HIV-1 infection, and to discover new agents that can efficiently inactivate NF-κB and HIV-1 transcription. Previously we demonstrated that DING p27SJ/p38SJ can suppress HIV-1 gene transcription through its interaction with HIV-1 Tat [1,7]. Further, we showed that DING p27SJ and p38SJ exhibit phosphatase activity [5]. Thus, here we investigated the possibility that DING proteins p27SJ and p38SJ can act *via* the de-phosphorylation of the p65 NF-κB transcription activator. In this study, we report that DING proteins can inhibit HIV-1 infection in cells, and activation of NF-κB complex through direct hypo-phosphorylation of p65, and downregulate LTR gene transcriptional activity.

## Materials and Methods

### Infection of peripheral blood mononuclear cells (PBMC) with HIV-1

Purified PBMC were prepared from a buffy coat after Ficoll-Hypaque isolation, and were maintained in modified RPMI media with 10% (v/v) Fetal Bovine Serum (FBS) supplemented with human recombinant IL-2 (20 IU/ml) following treatment with phytohemagglutinin (PHA; 5 μg/ml; Sigma) for 48 h. PBMC were infected with the HIV-1 JR-FL strain. Fifty ng of p24-containing virus stock were added to every 10^6^ cells. Cells were incubated with virus stock in a small volume of serum-free medium for 2 h at 37°C. The cells were washed two times with PBS, and then fresh medium was added. Prior to infection cells were transfected with DING expressing plasmids or treated with 50 or 250 ng of DING p38SJ. Two μl of lysis buffer were collected every 3 days. In parallel, control uninfected cells or cells infected with heat-inactivated HIV-1 for 10 minutes at 65°C, were prepared six days post-HIV-1-infection. Cells and supernatants were harvested and analyzed by ELISA, MTT and toxicity assay as described below. Infection of PBMC cells with monocyte/macrophage-tropic SF162 HIV-1 strain was performed as explained above, and lysates were used for p24 ELISA (with infection efficiency of approx 200 ng/ml of p24).

**U97 cells** were transfected first with DING p38SJ expression plasmids, and were then infected with SF-162 HIV-1 strain, 24h post-transfection, and analyzed on day 5 post-infection, while **T cells** were analyzed on day 7 post-infection. **Monocytes** and **U1** cells were infected with SF-162 (with approx 300 pg/ml and 15,000 pg of p24 accordingly).

### Treatment of human fetal microglial cells with anti-retroviral drugs

Microglial cells were plated in 60 mm dishes (200,000 cells), then transfected with DING p38SJ, DING p27SJ or pCDNA6 control plasmids. Forty eight hours post-transfection, cells were infected with HIV-1 SF162 strain (0.25 pg of p24/cell) for 4 hours. More medium was added for overnight incubation. Next day, cells were washed three times with PBS, and fresh medium was added, followed by the incubation with 10 μM of Tenofovir (TFV) and Emtricitabine (FTC) antiviral drugs alone or in combination. Fifty six hours later, treatment was repeated, then supernatants were harvested and fresh medium was added, and p24 ELISA was performed according to the manufacturer’s protocol (Advanced Bioscience Laboratories, Rockville, MD).

### p24 ELISA

Approximately 1 × 10^6^ PBMC were infected with HIV-1 JF-RL or SF-162 strains. Six to seven days post-infection, supernatants were collected and analyzed for the presence of HIV-1 p24 by ELISA using p24 ELISA Assay kit (Advanced Bioscience Laboratories, Rockville, MD). The assay was measured at 450 nm colorimetrically to indicate the concentration of Gag-p24 in samples. The concentrations of HIV1 p24 were measured in triplicates. Data were calculated from the HIV-1 p24 standard curves and normalized for sample dilution. Values are shown as graphs (mean ± SD, n=9). Each infection was repeated minimum of 3 times, in triplicates for each sample.

### Cell culture

U-87 MG inducible cell line that expresses DING p27SJ under doxycycline treatment was used in western-blot studies. U-87 MG glioblastoma astrocytoma cells with high efficiency of transfection were purchased from American Type Culture Collection (ATCC, Manassas, VA). Cells were cultured in DMEM with 10% FBS (Life Technologies, Inc.), 100 units/ml penicillin and 10 μg/ml streptomycin) at 37°C. DING-p27SJ inducible cell line in U-87 MG was created based on the pTetOn System (BD Biosciences Clontech, Palo Alto, CA) as we aspreviously described [1]. DING-p27SJ expression was induced by 1 mg/ml doxycycline (Dox) treatment of cells for 48 h.

### Plasmids

HIV-LTR (−476/+66) was cloned by PCR amplification of the LTR and ligation into *Hind*III–*Kpn*I site of pGL3 vector (Promega Corp., Madison, WI) [1]. CFP-tat plasmid was cloned by insertion of a *Bam*HI-*Eco*RI tat fragment from CMV-tat into *BglII-EcoR*I-pECFP-C1 plasmid [1]. We verified the sequence of plasmids by DNA sequencing. Oligonucleotides were made by Oligos Etc (Wilsonville OR).

### Cloning of cDNA encoding full-length DING p38SJ

DING p38SJ was cloned using RT-PCR and forward primers designed from the known DING p27SJ sequence and reverse primers from the Pseudomonas DING gene. Two fragments (1-788 and 788-1179) were then combined, and full-length cDNA was created to encode the 38 kDa DING p38SJ (364 amino-acids, GenBank #: AAW57408.2). DING p38SJ cDNA was then cloned into pcDNA6 expression vector (Invitrogen) [2].

### Transfections

Lipofectamine 2000 was used for transient transfection experiments, according to the manufacturer’s recommendations, using U-87 MG cells. In brief, 2 × 10^5^ cells in 60 mm plates were transfected with 0.5 μg of HIV-LTR-luciferase reporter plasmid, with or without 1 μg of DING-p38SJ and its deletion mutants, CFP-Tat or pECFP-p65. Experiments were controlled with a promoter, pCMV or pECFP-C1 plasmids to equalize the DNA amount in each sample. Cell extracts were collected at 36 h post-transfection, Cell extracts were collected at 36 h post-transfection and used for a Luciferase assay. Samples were assayed in triplicates.

### RNA preparation

RNA was isolated from U-87 MG cells or primary microglial cells, transfected with DING p27SJ and infected with HIV-1, with the RNAqueous^®^ total RNA Isolation Kit (Ambion, Austin, TX, USA). One μg of RNA was used to generate cDNA with reverse transcriptase (Roche Molecular Biochemicals, IN). The cDNA amplification with 28 cycles of PCR was performed using *Taq* DNA polymerase. PCR products were studied by DNA gel electrophoresis in 1.5% agarose gel.

### RT-PCR

In RT-PCR assays, the One-Step RT-PCR System (the Superscript III with Platinum *Taq* Invitrogen, Carlsbad, CA, USA) was used. To amplify the p65 cDNA, primers specific to p65 gene and three-step cycling, were used: 1) pre-denaturation at 55°C for 30 minutes; 2) 40 cycles of PCR amplification for 15-second, denaturing at 94°C, 30-second annealing at 60°C and 1 minute extension at 68°C; and 3) final extension for 5 minutes at 68°C. PCR products, 0.8 kb and 0.2 kb DNA were studied by DNA gel electrophoresis in a 1.5 % agarose gel.

### Northern blot assay

Ten μg of total RNA isolated from primary microglial cells, were analyzed by electrophoresis on a 1.2% agarose gel with 0.4% formaldehyde, 1 × Morpholinepropanesulfonic acid(Mops). RNA was transferred to Hybond-N nylon membrane (Amersham, Piscataway, NJ) as previously described [7,54]. To detect HIV-1 LTR and DING p27SJ RNAs, the membranes were hybridized with [32P]-labeled LTR DNA probe or DING p27SJ probe (800 bp). For the detection of GAPDH RNAs, the filters were probed with a PCR-amplified and [32P]-labeled GAPDH RNA, a housekeeping gene used as an internal control fragment. Radiolabeled DNA probes were prepared using Random Primed DNA Labeling Kit (Roche Molecular Biochemicals, Indianapolis, IN). Unincorporated radionucleotides were removed using MicroSpin™G-50 columns (Amersham). Housekeeping gene GAPDH was used as an internal control.

### Cell treatment and transfection

As mentioned above, for transfection assays, 2 × 10^5^ cells were cultured in 6 × well plates for 24 hours. Then, for p65 NF-κB stimulation, cells were kept in FBS free medium for 2 hours prior to Phorbol 12-Myristate 13-Acetate (PMA) treatment (10 nM) for 2 hours.

### Preparation of protein extracts

Cell lysates were prepared by washing of cells with cold PBS and incubating in lysis buffer (Sigma). After 5 min centrifugation at 4°C, cell debris was removed and fifty micrograms of protein lysates were heated at 95°C for 10 min with Laemmli sample buffer, then separated by 10% SDS-PAGE.

### Nuclear and cytoplasmic extraction

Cell fractionation assay was performed using the NE-PER kit (Pierce Biotechnology, Rockford, IL) to isolate nuclear and cytoplasmic protein fractions. The cells were lysed in NE-PER lysis buffer supplemented with protease inhibitor cocktail (Halt; Pierce Biotechnology). Protein concentration was determined by Bradford assay (Bio-Rad), and 10 μg of nuclear protein lysate and 20 μg of cytoplasmic protein lysate were applied on by sodium dodecyl sulfate-polyacrylamide gel (SDS-PAAG) electrophoresis (SDS-PAGE) (4%-20% gradient gels; Bio-Rad).

### Western-blot analysis

**Figure 1:**
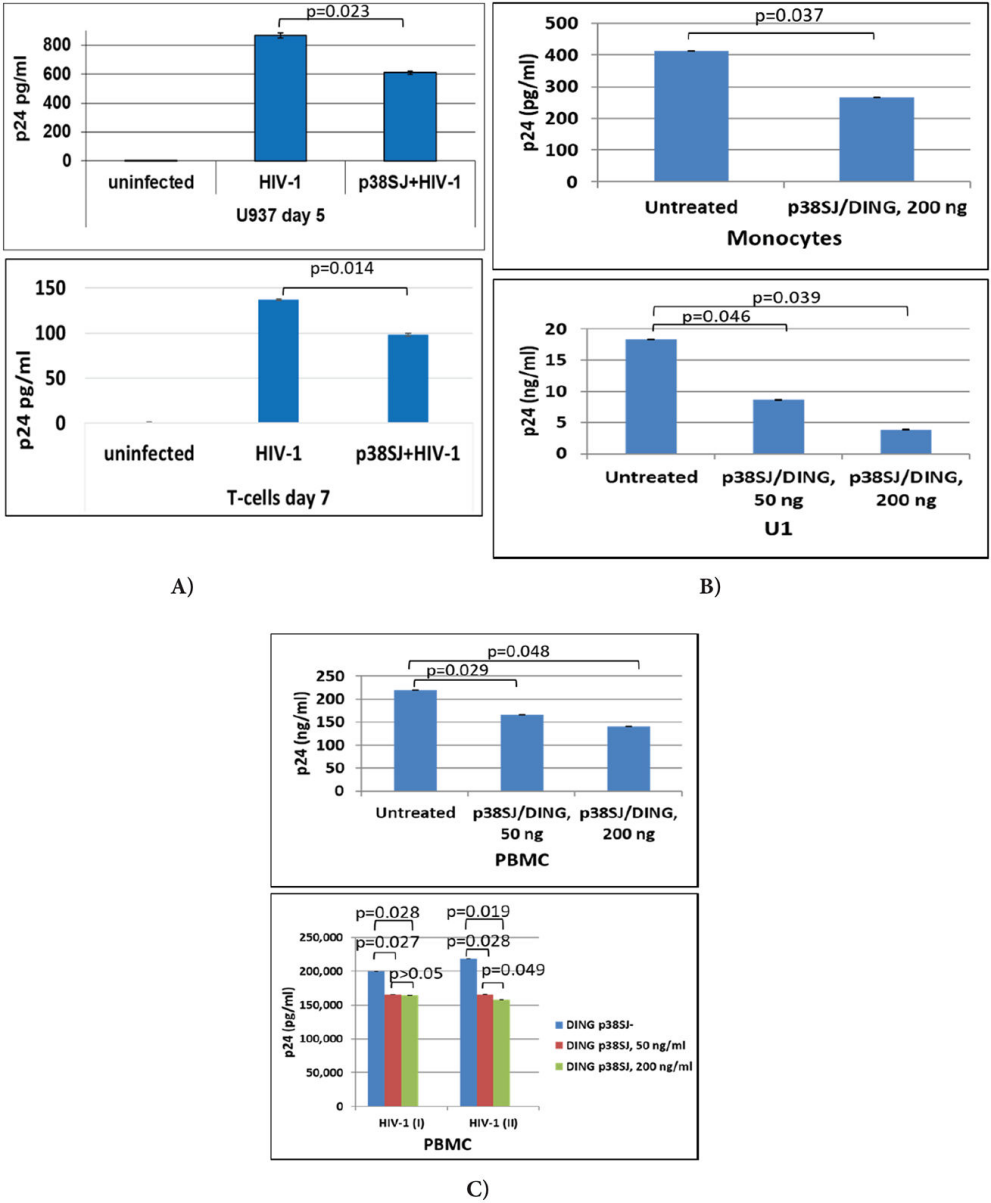

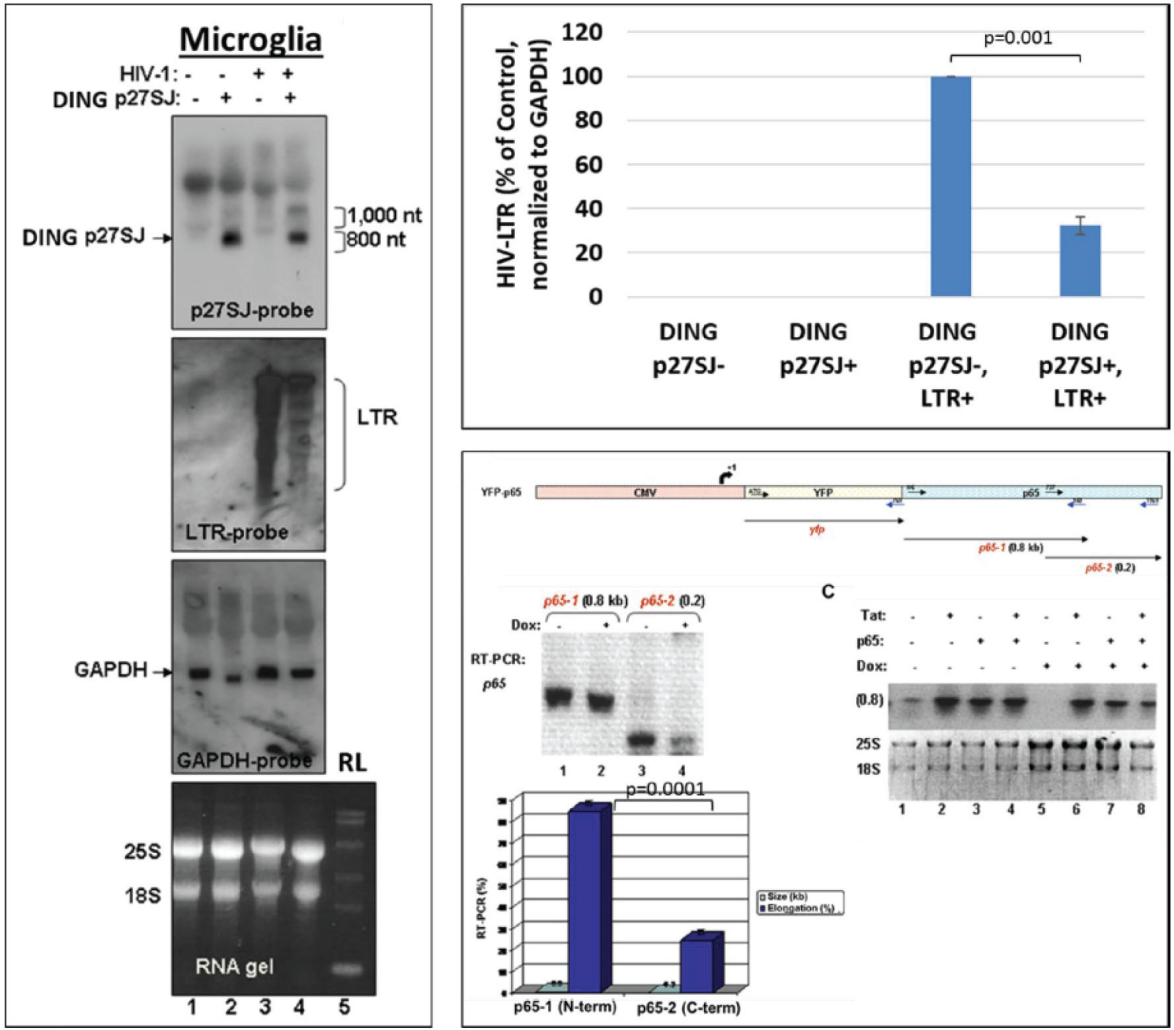

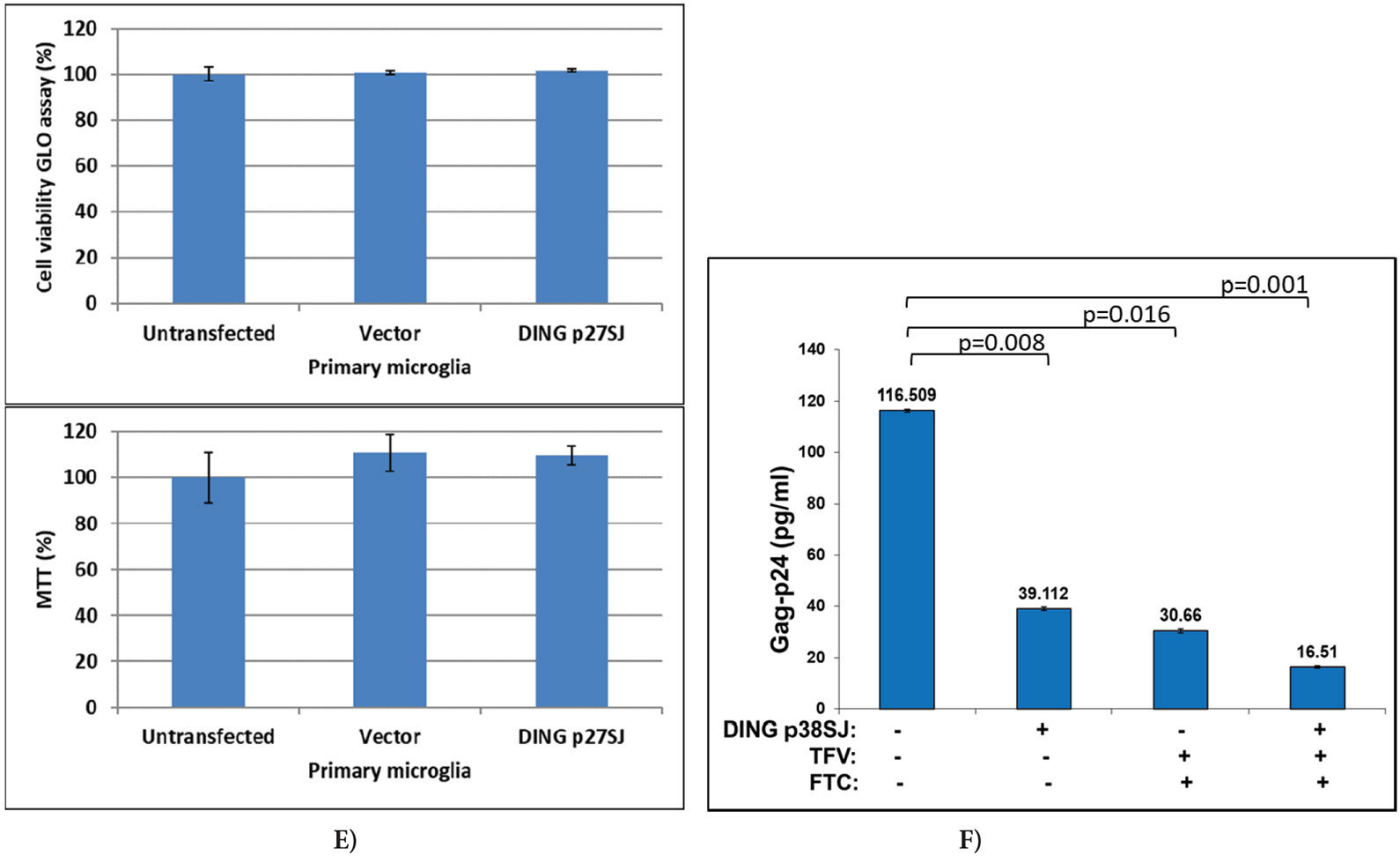
DING inhibits HIV-1 infection and replication. Suppression of HIV-1 by DING p38SJ and DING p27SJ in U937 and T-cells (A) in monocytes and U1 cells (B) and in PBMC (C) assayed by ELISA. Infection of PBMC cells with monocyte/macrophage-tropic SF162 HIV-1 strain was performed in two independent experiments and the concentrations of HIV-1 p24 were measured in triplicates, interpolated from the HIV-1 p24 standard curves and corrected for sample dilution. The values are graphed (mean ± SD, n=3) **(D)** Inhibition of HIV-1 infection in microglia by DING. DING p27SJ inhibits HIV-1 SF-162 in human fetal microglia shown by northern blot analysis (left panels). Primary human microglial cells were transfected with DING p27SJ plasmid followed by infection with HIV-1 SF162 strain. After 5 days, total RNA from HIV-1 infected cells was prepared and analyzed by northern blot using DNA probes derived from the HIV-1 genome, DING p27SJ, and housekeeping GAPDH (left panels). Total RNA was separated on a denaturing formaldehyde-agarose gel and hybridized to labeled LTR, DING p27SJ and GAPDH probes. The integrity of the RNA preparation is shown on the bottom panel. The positions of the 18S and 28S RNAs in the gel are indicated. Bands were analyzed by densitometry (top right panel), demonstrating up to 70% suppression of LTR. Effect of DING p27SJ on p65 NF-κB gene transcription and elongation (bottom right panels). Schematic representation of the plasmid, carrying p65 gene fused in frame with *yfp* gene under control of CMV promoter, used in transcription elongation studies shown on the top. The sites of the primers used in the RT-PCR elongation assays are specified as the positions of two amplicons. Inhibitory effect of DING on p65 and YFP transcription/elongation was studied in RT-PCR assays for two amplicons. The image of the ethidium bromide-stained gel was inverted using Adobe Photoshop for clarity of presentation. The longer is transcript (compare lane 3 with lane 1) and the further is a primer from transcription initiation start, the stronger is inhibitory effect of DING on elongation of the transcript (compare lanes 4 and 3, with 2 and 1). Bottom panel demonstrates a graphical representation of the elongation assay as bars (bottom panel). Level of yfp-p65 RNA expression and integrity and accuracy of RNA loading was examined by agarose gel electrophoresis and ethidium bromide staining of RNA gel for RNA samples from cells without (lanes 1 to 4) or with DING (lanes 5 to 8), transfected with p65 and Tat in various combinations. The image of the gel was inverted using Adobe Photoshop for clarity of presentation. **(E)** Cell viability assays demonstrating cell proliferation/toxicity of p27SJ in primary culture of microglial cells, transfected with CMV-p27SJ or control empty vector, pCDNA6. After 48 hours, cells were harvested and assayed by Cell Viability GLO assay (top) and MTT assay (bottom). As shown, the expression of DING p27SJ had no major effect on the viability of primary culture of microglial cells as tested by GLO and MTT assays. Data were collected from three independent experiments with triplicated samples. Error bars represent SD. (F) HIV-1 p24 ELISA for human fetal microglial cells transfected with DING p38SJ or the vector pCDNA-C1. Forty eight hours post-transfection, cells were infected with HIV-1 SF162 strain (0.25 pg of p24/cell) for 4 hours, and then medium was added for overnight incubation, followed by the treatment with 10 mM of Tenofovir (TFV) and Emtricitabine (FTC) antiviral drugs alone or in combination. Fifty six hours later, treatment was repeated, and p24 ELISA was performed. The level of HIV-1 p24 (bar 1) dropped from 116 pg/ml to 39.11 pg/ml in the presence of DING p38SJ (bar 2), while the treatment with both drugs dropped p24 level to 30.66 pg/ml (bar 3). Treatment of cells with DING and both drugs lead to a significant decrease of p24 level from 30.66 pg/ml to 16.51 pg/ml (bar 4), suggesting an efficient suppression of HIV-1 by combinatory drug use. Data were collected from 3 independent experiments with triplicates for each sample. Error bars are for SD.

**Figure 2:**
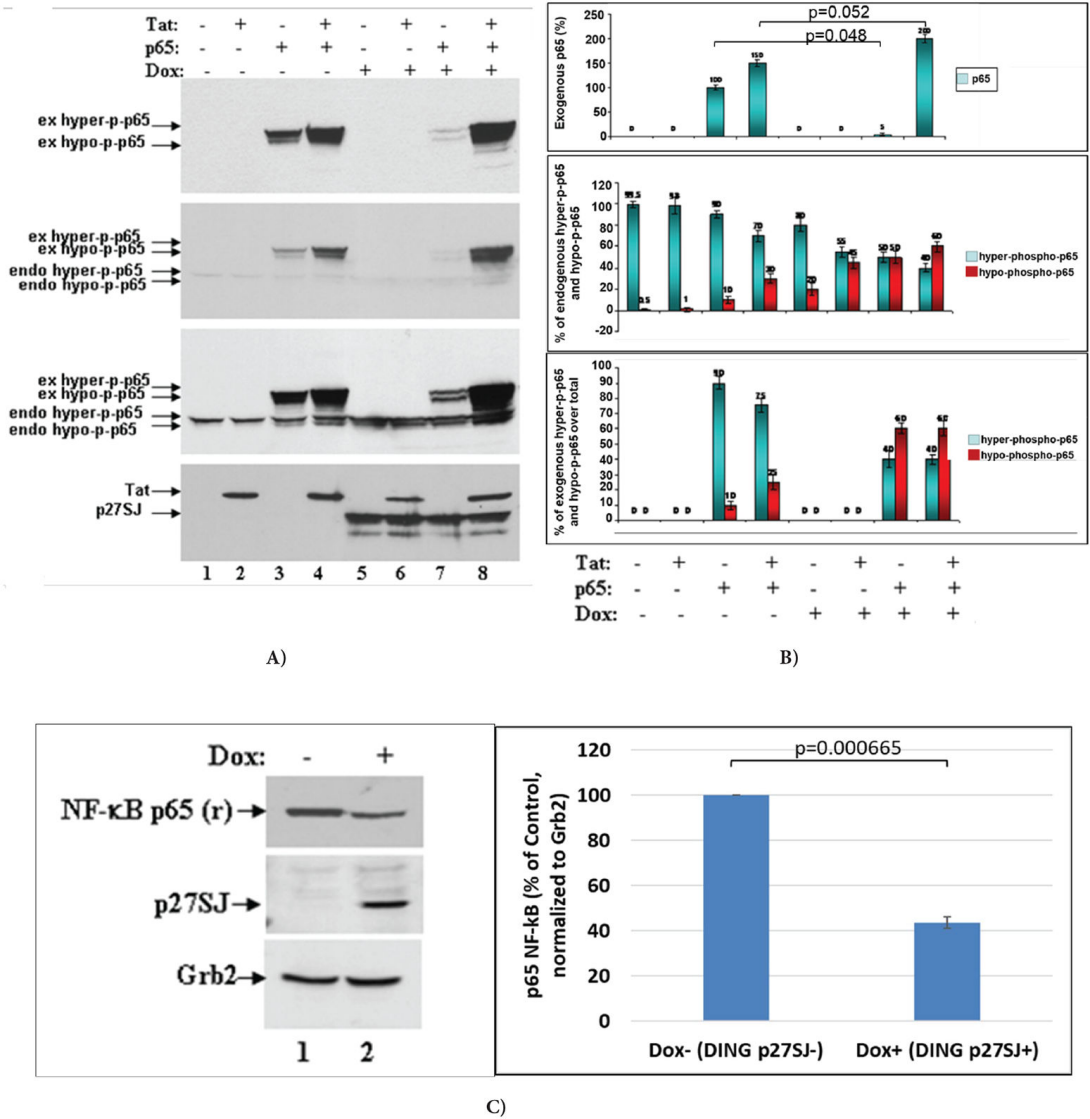
Inhibition of p65 phosphorylation by DING p27SJ. **(A)** Western blot analysis of extracts from the cells expressing DING p27SJ induced by doxycycline (Dox+, lanes 5-8) or without DING p27SJ (Dox−, lanes 1-4). To assay the phosphorylation of p65 in cells expressing Tat and/or DING p27SJ, DING p27SJ inducible cells were transfected or untreated with exogenous Tat or p65 NF-κB expressing plasmids. Cellular extracts were subjected to western blot using antibodies to hyper-phosphorylated or total p65 NF-κB (panels 1-3), Tat and DING p27SJ (panel 4). Images of a blot with different exposure time are shown (panels 2-3). Grb2 in served as a loading control. **(B)** Levels of exogenous and endogenous hyper-phosphorylated and hypo-phosphorylated p65 were analyzed by densitometry and shown in %. Error bars present SD from three readings. Experiments were repeated 2 times. **(C)** Western blot analysis of extracts from cells treated or untreated with Dox to induce DING p27SJ. The expression of endogenous p65 NF-κB and DING proteins in p27SJ cells was confirmed in western-blot assay using relevant polyclonal antibodies. Grb2 served as a loading control. Error bars on the graph demonstrate SD from three readings.

**Figure 3:**
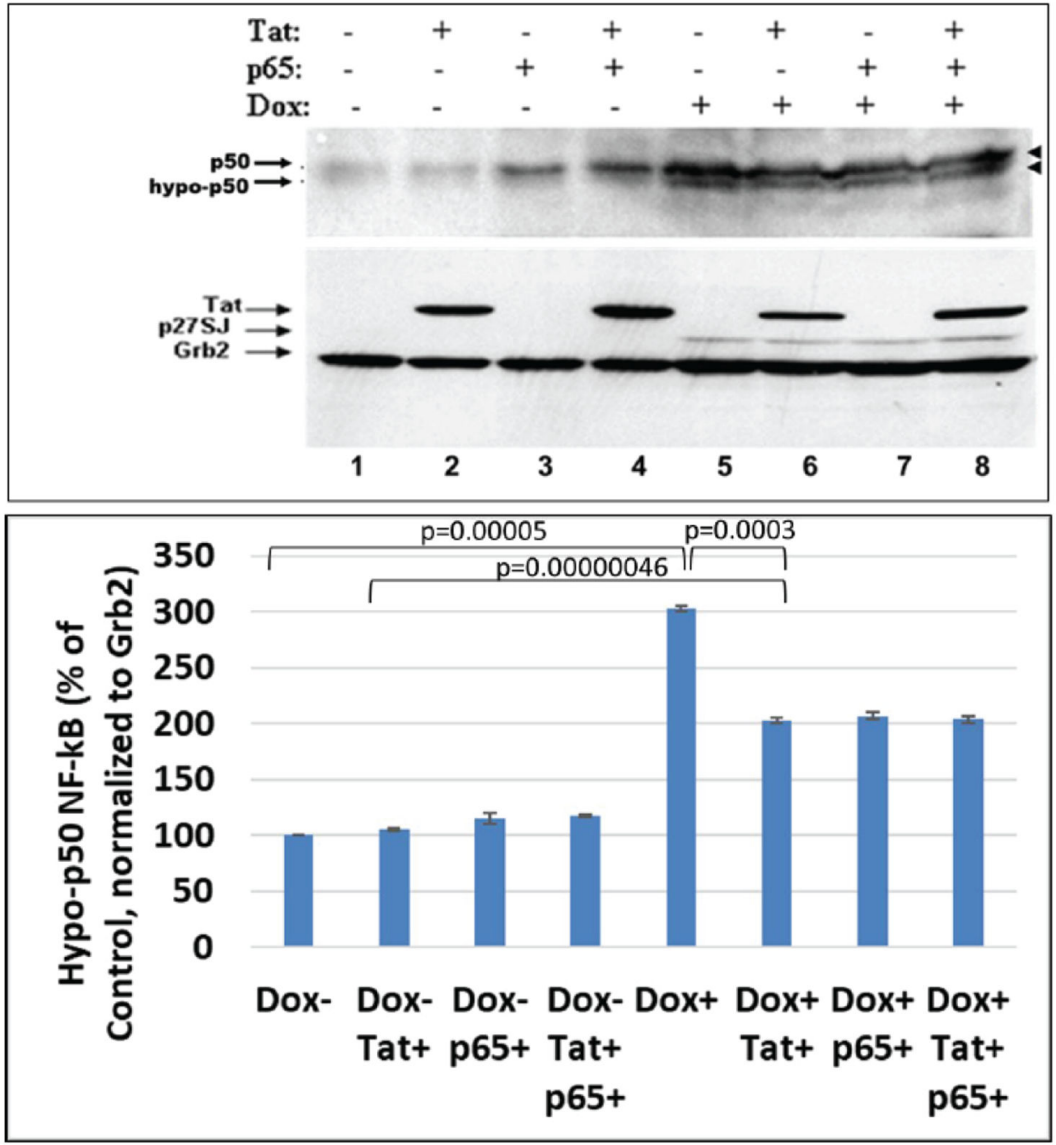
Effect of DING p27SJ on dephosphorylation of NF-κB p50. Western-blot analysis of extracts expressing DING p27SJ demonstrates similar pattern of dephosphorylation of p50, p65 partner in NF-κB heterodimer (lanes 5-8, top panel). Grb2 served as a loading control (bottom panel). Double arrows (top panel) show hyper-and hypo-posphorylated p50. Densitometry for hypo-phosphorylated p50 is presented on the bottom graph. Error bars represent SD from three readings.

**Figure 4:**
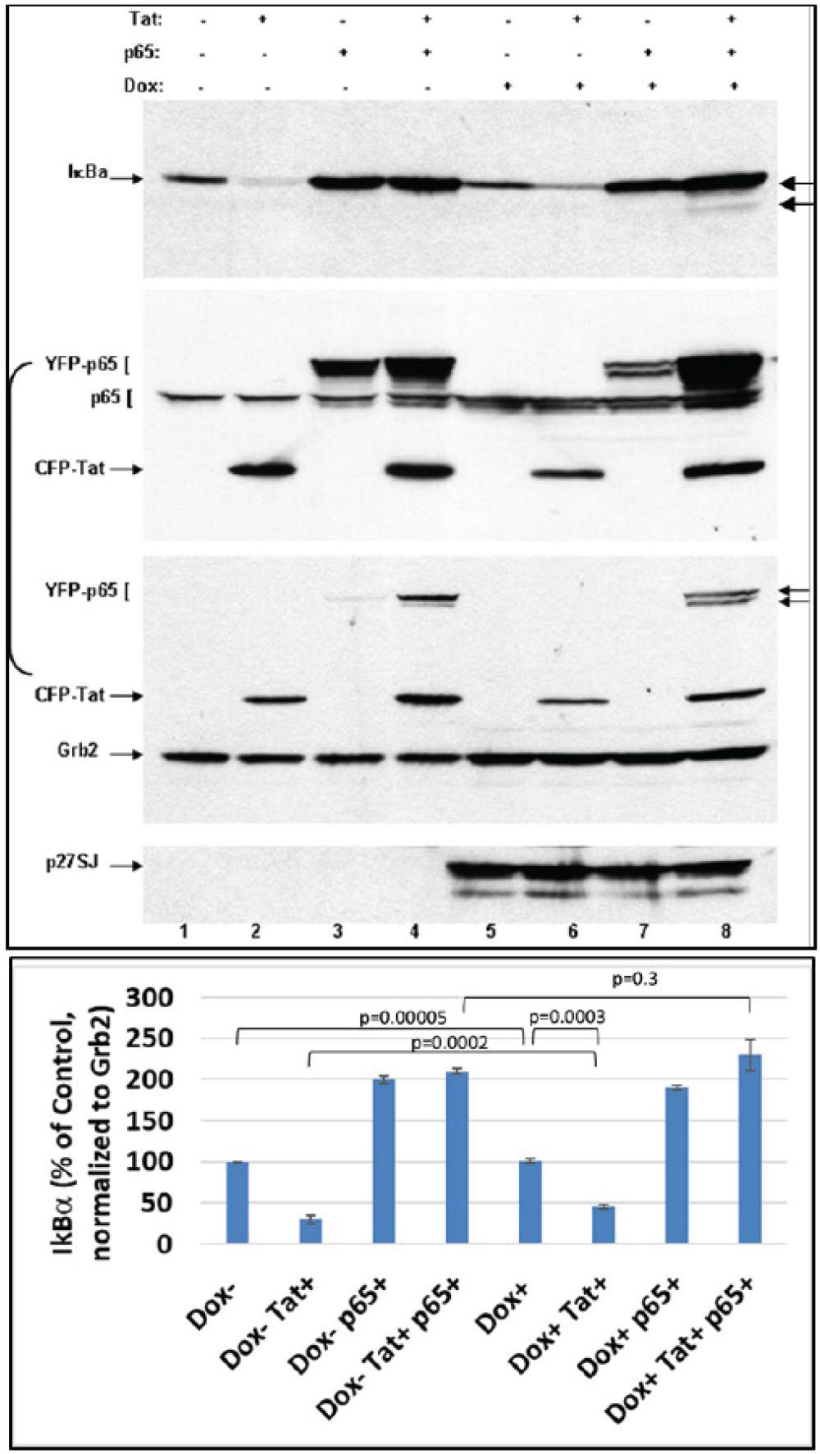
Dephosphorylation of NF-κB complex by DING p27SJ is I-κB independent. Western blot assay shows that the dephosphorylation and inactivation of p65/p50 NF-κB in DING expressing cells is IκBα-independent, as drastic changes in NF-κB levels (2^nd^ and 3^rd^ panels) are not affected by I-κBα expression (lanes 4 and 8, top panel). NF-κB p65 changes are associated with the expression of DING. While I-κBα is expressed at higher level in the presence of p65 (lanes 3, 4 and 7, 8), it is more stabilized by DING and reverses Tat-associated I-κBα inhibition (lanes 2 and 6). Top panel shows I-κBα level. Second panel demonstrates p65 (both, exogenous YFP-p65 or endogenous p65) and Tat levels. Third panel shows Grb2 level that was used as a loading control. Arrows point at the hyper- and hypo-phosphorylated p65. Bottom panel demonstrates induction of p27SJ upon Doxycycline treatment. Error bars represent SD fom 3 readings.

**Figure 5:**
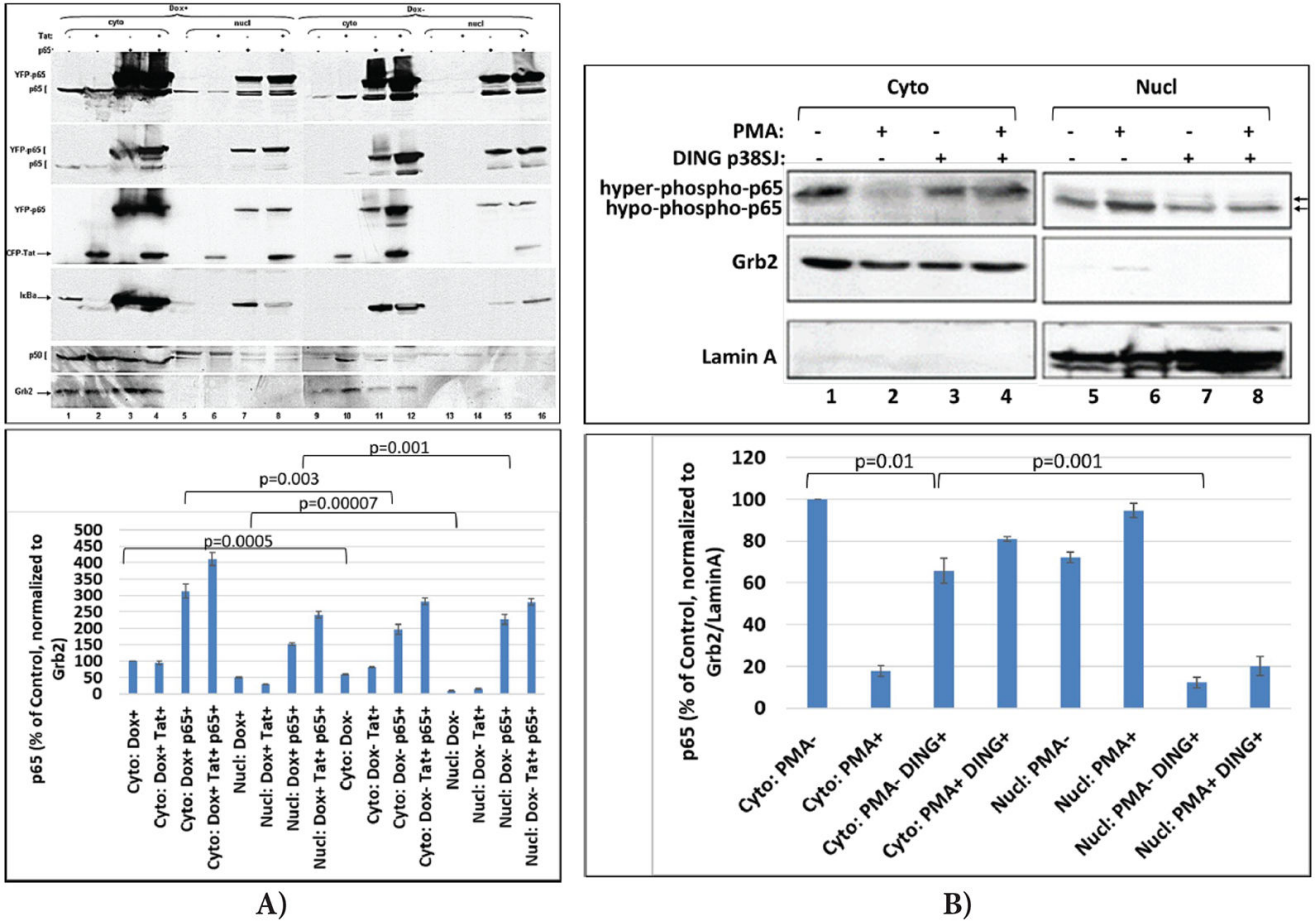
DING interferes with NF-κB trafficking. **(A)** DING 27SJ disrupts p65 NF-κB translocation to the nucleus. Cell fractionation and western-blot assay to demonstrate the effects of DING p27SJ on the translocation of p65/p50 from cytoplasm to nucleus. Panels 1 and 2 demonstrate more cytoplasmic than nuclear accumulation of p65 in DING (Dox+) cells (lanes 1 to 4 compare with 5 to 8). Panel 5 shows similar intense cytoplasmic localization of p50 (lanes 1 to 4 compare with 5 to 8). p65 overexpressing cells (lanes 3 and 4 compare with 7 and 8) and Tat overexpressing cells (panel 3, lanes 2 and 4 compare with lanes 6 and 8) in DING p27SJ-expressing cells (lanes 1-8). In Dox− cells such effect is not drastic (lanes 9-16). Dephosphorylated p65 was detected more intensively in the nucleus of DING p27SJ-expressing cells (compare lanes 7 and 8 with 15 and 16, top panel). Panel 4 shows p65 inhibitor, IκBα, with more accumulation in cytoplasm of DING p27SJ-positive cells (compare lanes 3 and 4 with lanes 11 and 12), although it was found also in nucleus. Panel 6 demonstrates loading controls for cytoplasmic Grb2 (lanes 1-4, and 9-12) and nuclear Lamin A (lanes 5-8, and 13-16) fractions. **(B)** Effect of DING p38SJ on the PMA-induced activation of NF-κB p65 and its translocation into nucleus. Western blot assay was performed with cytoplasmic and nuclear lysates from cells transfected with full length DING p38SJ, and induced with PMA. Increased cytoplasmic accumulation for p65 is shown (lanes 3 and 4 compare with lanes 1 and 2) in DING p38SJ-expressing cells. More PMA-induced p65 is found in the nucleus of cells lacking DING protein. Error bars represent SD from 3 separate readings.

Protein samples were transferred to supported nitrocellulose membranes at 40 mAmps. Detailed procedures were described previously [1]. To visualize proteins, the enhanced chemiluminescence detection system, ECL+ was used (GE Healthcare, Piscataway NJ). The Grb2 levels shown in figures 1-4 and Grb2 and Lamin A shown in figure 5 served as gel loading controls.

### Antibodies

Anti-DING p27SJ and anti-p38SJ (rabbit polyclonal antibodies) were generated and purchased from Lampire Biological Laboratories, Inc (Pipersville, PA). Anti-p65 (F-6), Grb2, and Lamin A antibodies were obtained from Santa Cruz Biotechnologies, Inc. (Santa Cruz, CA). Antibody for CFP-Tat (Living Colors) was from BD Biosciences Clontech. Expression of the YFP-fusion proteins was confirmed using Living Colors monoclonal antibody (BD Biosciences Clontech). Anti-Myc-tag monoclonal antibody was obtained from Invitrogen.

### Luciferase reporter assay

U-87 MG inducible cells were transfected with LTR-luciferase construct in the absence or presence of plasmid-expressing Tat, p65 NF-κB, then incubated with Doxycycline for 24 hours for DING p27SJ expression. An equal aliquot of each reaction was assayed for luciferase activity using the Dual-GloTM Luciferase Assay System (Promega) according to the manufacturer’s recommendations. All reporter assays were performed at least 3 times in triplicates for each sample. Relative luciferase units were converted into fold activity, and presented graphically. Luminescence was recorded on a Turner Designs Luminometer TD-20/20 (Promega). Data were analyzed using Excel software.

### Methylthiazoletetrazolium (MTT) assay

For the MTT cell proliferation assay, we used microglial cells and MTT kit (Roche, Basel, Switzerland). Cells were plated onto 96-well plates in triplicates, and in three sets at a density of 15,000 cells/well. Cells were transfected with DING p27SJ. Twenty four hours following transfection, 10 μl MTT (5 mg/ml) were added to the wells (final concentration, 0.5 mg/ml) for 4 h, and the reaction was stopped by the addition of 100 μl of solubilization solution. Viable cells with active mitochondria cleave the tetrazolium ring into a visible dark blue formazan reaction product, which was quantified by spectrophotometry in a microplate reader at 570 nm with a reference wavelength of 650 nm. The relative cell viability (% of control) for each sample was determined as the ratio of average absorbance for transfectd cells to that for untransfected or transfected with an empty vector.

### The CellTiter-Glo™ luminescent cell viability assay

This assay requires ATP, a co-factor of the luciferase reaction, as an indicator of metabolically active cells. Since the luciferase reaction requires ATP, the produced luminescence is proportional to the amount of ATP present, an indicator of cellular metabolic activity. To perform the assay, CellTiter-Glo™ Reagent (Promega, Madison, WI, USA) was added in a volume equivalent to the amount of medium in which the cells were plated. After 2 minutes mixing and 10 minutes incubation, the emitted luminescence was measured using a plate reading Turner Designs Luminometer TD-20/20 (Promega). Data were analyzed using Excel software.

### Statistical analysis

Statistical analysis was performed using SPSS (IBM Corp., released 2017), IBM SPSS Statistics for Windows, Version 25.0. Armonk, NY), and EXCEL, and ImageJ. Mean values were compared using the Student’s t-test with Bonferroni correction for multiple comparisons, where relevant. Statistical significance was defined as p<0.05. Data were exported to Microsoft EXCEL for further statistical analysis.

The p-value was calculated based on a Student’s t-test of the triplicate values (normalized to internal controls) for each sample in each control group and test group comparison from 2-3 experiments in triplicates. The p-value calculation used was based on parametric, unpaired, two-sample equal variance, two-tailed distribution-a method widely accepted in scientific literature. Both Groups in each pairwise comparison must contain at least 3 samples for the software to calculate p-values for that comparison.

## Results

This study was aimed to unravel the mechanism of HIV-1 inhibition by DING p27SJ and DING p38SJ through de-phosphorylation and inactivation of p65, forced translocation of p65 to the cytoplasm, and suppression of p65 binding to the HIV-1 LTR. First, we studied if DING inhibits HIV-1 infection in cells.

### DING p27SJ and DING p38SJ inhibit HIV-1 infection

To investigate if DING proteins inhibit HIV-1 replication in several human cell types, comprehensive infection assays were performed using PBMC, U937 (one of only a few human lines still expressing many of the monocyte like characteristics), primary microglia, T-cells, monocytes, and U1 promonocytic cells for infection with two HIV-1 strains, JR-FL and SF-162 (Figure 1). U937 and T-cells were first transfected with full-length DING p38SJ prior to HIV-1 infection. HIV-1 Infection was strongly inhibited by DING in both U937 and T-cells at day 5 or day 7 post-infection, confirmed by HIV-p24 levels (Figure 1A). We found similar suppressive effects on HIV-1 replication in monocytic and pre-monocytic (U1) cells when incubated with DING p38SJ using biologically relevant concentrations. Treatment of cells with 50 ng/ml of DING protein caused up to 50% inhibition in U1, while using 200 ng/ml led to up to 80% inhibition in U1, and up to 50% inhibition in monocytes, as shown in figure 1B. Suppression of HIV-1 by DING p38SJ was also observed in PBMC treated with two concentrations of DING protein (Figure 1C). Results from two independently repeated experiments are shown on the bottom panel of figure 1C. DING p27SJ was also able to inhibit HIV-1 SF-162 strain in microglia (Figure 1D). HIV-LTR RNA expression was inhibited in infected primary microglial cells expressing DING, compared to cells transfected with empty plasmid only, shown by northern blot assay (Figure 1D, left panels), and by densitometry (top right panel), demonstrating up to 70% suppression of LTR.

Thus, DING inhibits HIV-1 replication and LTR gene expression/ transcription. To show whether DING can inhibit also inhibit expression of the transcription factor, p65-NF-κB, an activator of LTR, we performed transcription elongation assay.

### Effect of DING p27SJ on p65 gene transcription and elongation.

The inhibitory effect of DING on p65 NF-κB transcription/elongation was studied using RT-PCR assays (Figure 1D, bottom right). Schematic representation of the plasmid, carrying p65 gene fused in frame with yfp gene under control of CMV promoter, used in transcription elongation studies is shown on top. Primers used in elongation assays are specified. Quantification of the bands shown in gel demonstrated that the effect of the DING p27SJ inhibition correlated with the position of the reverse primers, like with closest (p65-1) there was slight inhibition (12%) and with the farther primer (p65-2) there was a stronger inhibition (75%). Numbers are shown on the graph. We suspected that DING p27SJ can also inhibit also transcription elongation of YFP-p65. Results from elongation assay demonstrate that the longer is transcript (compare lane 3 with lane 1) and the further is a primer from transcription initiation start, the stronger is the inhibitory effect of DING on elongation of the transcript (compare lanes 4 and 3, with 2 and 1). Integrity and accuracy of RNA loading is demonstrated in RNA gel for RNA samples from cells with (lanes 5 to 8) or without DING (lanes 1 to 4), transfected with p65 and Tat in various combinations. Importantly, DING protein did not have any toxic effects on cells, demonstrated by Cell Viability GLO and MTT assays (Figure 1E), suggesting its potential to be developed into a safe therapeutic drug for HIV-1 inhibition. Further, we compared inhibitory effects of DING on HIV-1 with two known antiviral drugs Tenofovir (TFV) and Emtricitabine (FTC), and found that both drugs suppress HIV-1 replication more efficiently when used in combination with DING protein (Figure 1FF). The level of HIV-1 p24 dropped from 116 pg/ml (bar 1) to 39.11 pg/ml in the presence of DING p38SJ (bar 2), while a treatment with both drugs dropped p24 level to 30.66 pg/ml (bar 3). Treatment of cells with DING and both drugs lead to a significant decrease of p24 level from 30.66 pg/ml to 16.51 pg/ml (bar 4), suggesting an efficient suppression of HIV-1 by combinatory drug use. Data were collected from 3 independent experiments with triplicates for each sample. Error bars are for SD.

Next, we studied if the post-transcriptional modifications and phosphorylation of the p65 NF-κB transcription factor can be inhibited by DING phosphatase.

### DING p27SJ inhibits p65 phosphorylation.

To study if the phosphorylation of p65 NF-κB is affected by the phosphatase activity of DING p27SJ, we used an inducible cell line that expresses DING under Doxycycline treatment, DING p27SJ-inducible cells. Cells were first transfected with p65 and Tat expression plasmids alone or in combination. Cells were then treated with or without doxycycline to induce DING p27SJ expression, and cell lysates were collected to perform western blot analysis. The role of DING protein on a function of endogenous or exogenous p65 NF-κB was studied in the absence or presence of Tat in the Dox-inducible DING p27SJ cell line. As shown in figure 2A, the co-expression of DING protein affected the levels of exogenous p65 NF-κB protein (compare Figure 1A lanes 3 & 7). The DING-mediated block of p65 NF-κB expression could be mitigated by the co-expression of HIV-1 Tat protein (Figure 2A, Lanes 4 & 8), while the expression of endogenous p65 NF-κB was unaffected by the presence of DING or Tat proteins (Figure 2A, lanes 5 & 6). In contrast, the phosphorylation of both endo- and exogenous p65 NF-κB proteins was blocked in the presence of DING and could not be rescued by the presence of Tat (Figure 2A, Lanes 5-8). The results of the western blot were confirmed by densitometry (Figure 2B), and the expression of p65 in cells was confirmed in western-blot assay, using polyclonal anti-p65 antibody (Figure 2C, lane 2). The Grb2 protein (Figure 2C, bottom panel) was served as gel loading control. The densitometry of bands is presented on the bottom of each panel. Experiments were repeated 2 times, and each calculation was done in triplicates. Error bars demonstrate SD results of this presented in this experiment clearly suggest that DING protein blocked the phosphorylation of p65 NF-κB, and rendered the protein biologically inactive.

### De-phosphorylation of NF-κB p50 in the presence of DING p27SJ

Blots for figure 1 were used to re-blot with anti-p50 antibody. Data from Western-blot analysis of extracts expressing DING protein demonstrate similar inhibitory effect of DING on p50, a p65 partner in NF-κB heterodimer (Figure 3, lanes 5-8, top panel). Hypo-phosphorylated form of p50 is indicated by arrows. We found a similar pattern of inhibition of phosphorylation for both p50 and p65 in DING-expressing cells, a more hypo-phosphorylated p50 in DING p27SJ expressing cells.

### Inactivation of NF-κB complex by DING is I-κB independent

The p50/p65 subunits of NF-κB dimer are separated in the cytoplasm when in a complex with the I-κBα protein, and upon cell activation, the p50/p65 NF-κB dissociates from that complex. To determine whether the diminished phosphorylation of p65 NF-κB may be influenced by its association with the I-κBα, we performed western blot analysis of p50/p65 NF-κB expression and phosphorylation in DING p27SJ cells upon their exposure to doxycycline and co-expression of Tat. The results from western blot assay showed that the levels of IκB protein were the same in systems, where cells were expressing the exogenous or endogenous p65 NF-κB or Tat and did not correlate with the expression of DING protein (Figure 4). These data suggest that dephosphorylation and inactivation of p65/p50 NF-κB in DING-expressing cells does not depend on the I-κBα while, the reduction of phosphorylated NF-κB protein levels correlated positively with the expression of DING protein (Figure 4, lanes 4 and 8, top panel). Although-κBα is expressed at higher levels in the presence of p65 NF-κB (Figure 4, lanes 3, 4 and 7, 8), it was stabilized by DING and reversed Tat-associated I-κBα inhibition (Figure 4, lanes 2 and 6). In addition, an extra band of dephosphorylated form of IκB was detected only in the DING-expressing cells transfected with Tat and p65, while dephosphorylated IκB was not seen in Dox− cells (Figure 4, lanes 4 and 8).

### DING interferes with NF-κB nuclear trafficking

Subsequently, we observed the compartmentalization of the p50/p65 NF-κB dimer in DING-p27SJ cells cells with or without doxycycline treatment. Following activation of DING protein expression, cells were subjected to fractionation into the nuclear and cytosolic compartments and western blot analysis. Cell fractionation assay demonstrates that translocation of p65/p50 NF-κB from cytoplasm to nucleus was affected by the presence of DING-p27SJ. We observed much higher cytoplasmic than nuclear accumulation of p50 NF-κB (Figure 5A, lanes 1 to 4 compare with 5 to 8) and p65 NF-κB proteins (lanes 3 and 4 compare with 7 and 8) in DING p27SJ-expressing cells. The cytosolic and nuclear fractions of cells that did not express DING protein (Dox− cells) showed Tat independent nuclear translocation of p65 NF-κB protein, which suggested that in DING expressing cells, HIV-1 may contradict the function of DING (Figure 5A, lanes 9-16). Dephosphorylated p65 was detected more intensively in the nucleus of DING p27SJ-expressing cells (compare lanes 7 and 8 with 15 and 16, top panel). The p65 NF-κB inhibitor, I-κBα, was more accumulated in the cytoplasm of the DING p27SJ-positive cells (compare lanes 3, 4 with lanes 11, 12). Interestingly, the results show that nuclear presence of I-κBα induces translocation of NF-κB to the cytoplasm from the nucleus [55]. I-κB alpha tightly regulates the transcriptional activity of NF-kappa B by retaining it in the cytoplasm in an inactive form. Our data suggest that nuclear appearance of I-κB in DING-expressing cells can cause p65 inactivation and translocation to the cytoplasm, and can play a secondary role in the effects of DING on NF-κB.

Next, we examined whether a similar disrupting effect on p65 translocation from cytoplasm into nucleus would be demonstrated by the full-length DING p38SJ, in addition to DING p27SJ, and whether this effect will be seen during NF-κB stimulation by PMA.

### Effect of DING p38SJ on the PMA-induced activation of NF-κB p65 and its translocation into nucleus

Cell fractionation assay in cytoplasmic and nuclear lysates from cells transfected with DING p38SJ and induced with PMA demonstrates more cytoplasmic accumulation of p65 (Figure 5B, lanes 3 and 4 compare with 1 and 2) in DING p38SJ-expressing cells. PMA-induced p65 was detected more intensively in the nucleus of control cells transfected with empty vector only (Figure 5B, compare lane 6 with lane 8, top panel). Thus, while PMA induces p65 translocation into the nucleus, DING protein reverses PMA effect on p65 and as a result, more p65 remains in the cytoplasm.

### DING p38SJ requires both, NF-κB and RPII for LTR suppression

**Figure 6:**
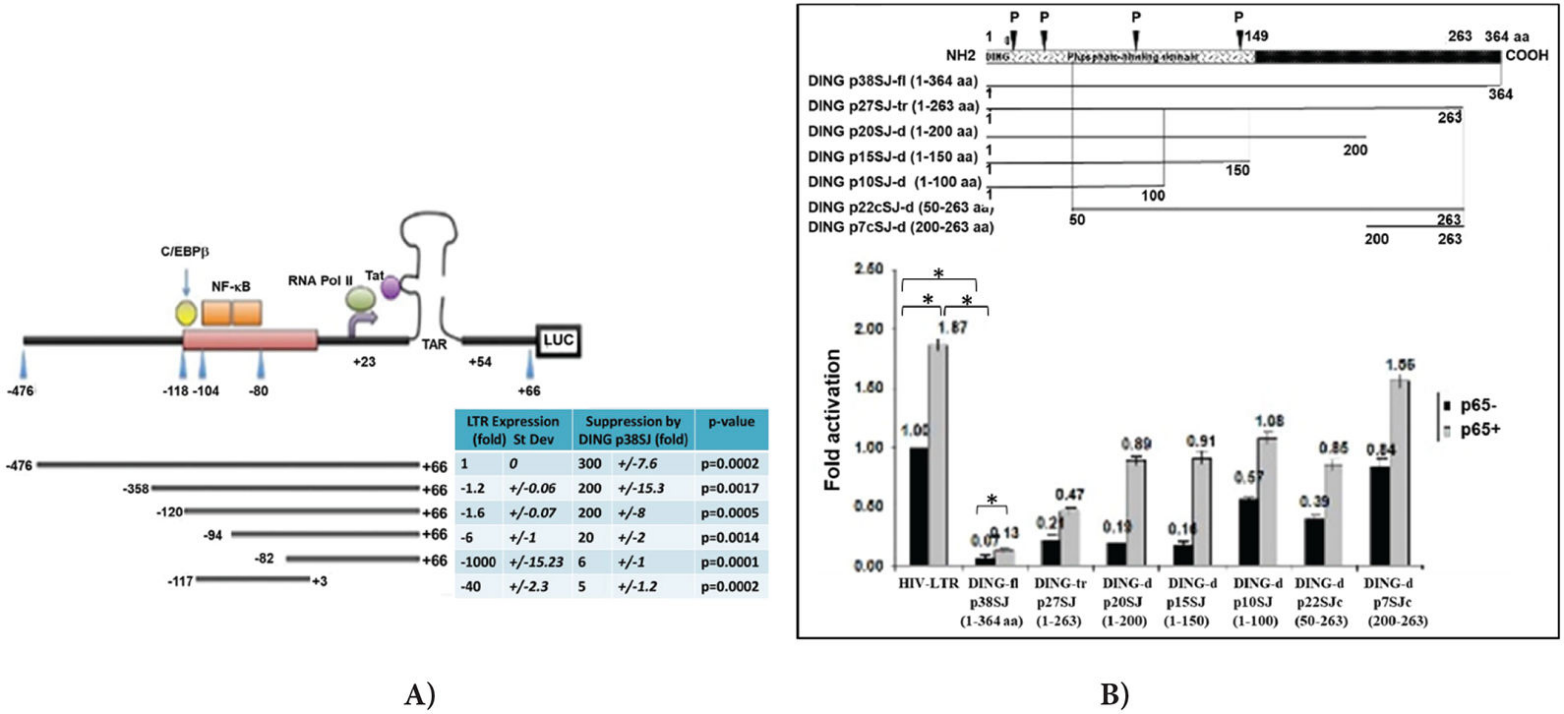
(A) DING requires both, NF-κB and RPII for LTR suppression. DING inhibits LTR transcription through NF-κB (−103/−80), RPII binding domains, and TAR region, observed in transcription in promoter/reporter assays. HIV-1 LTR deletion mutants were used in transcription assays with or without NF-κB (−103/−80), C/EBPβ (−118/−104), RPII (+23) and TAR (+22/+54) regions. The effects of DING p38SJ on the promoter activity of the HIV-1 LTR were examined using the HIV-1 LTR-luciferase reporter construct in the U-87MG cells. Top panel demonstrates a schematic representation of HIV-1 LTR constructs and important binding domains for viral and cellular transcriptional factors within its sequence: LTR deletion mutant (−117/+3) lacking both Tat binding site (TAR) and RPII, but still including NF-κB p65 (−104/−80), deletion of NF-κB (−84/+66), LTR mutants that partially lack NF-κB site (−94/+66) or completely lack NF-κB site (−82/+66). Bottom panel shows the effect of DING on HIV-LTR deletion mutants in Luciferase assay. Suppression of LTR transcription is presented in folds. Numbers in each column were normalized to the control in column 1 top raw (1 fold, or 100%), i.e., full-length HIV-LTR gene in the absence of DING p38SJ. Fold suppression of LTR mutants is presented in Column 1, with SD (Column 2). Suppression of LTR by DING is shown in Column 3 with SD (Column 4). P-values are presented in Column 5. **(B)** N-terminal region of DING p38SJ that contains phosphate-binding domain is important for the inhibition of p65-associated LTR transcriptional activation. Schematic representation of DING p38SJ deletion mutants (top). Effect of DING p38SJ on NF-κB associated LTR activation was studied in promoter/reporter assays, using various DING p38SJ deletion mutants (bottom). Suppression of LTR transcription is presented in folds. Numbers in each column were normalized to the control (1 fold, or 100%). Error bars demonstrate SD. Experiment The experiment was repeated 3 times with triplicate samples.

We performed Promoter-Reporter Luciferase assay to identify DING-responsive region within HIV-LTR gene, using LTR deletion mutants containing C/EBPβ (−118/−104), NF-κB, RNA Pol II and TAR binding domains. The effects of DING p38SJ phosphatase on the transcription activity of the HIV-1 LTR and its deletion mutants lacking either transcriptional binding domains were studied using the LTR-luciferase reporter plasmids and DING p38SJ-expressing cells (Figure 6A). In this experiment, transcription activity of the LTR deletion mutant (−117/+3) lacking both Tat binding site (TAR region, +22/+54) and RPII (+23), but still including NF-κB p65 (−104/−80), was suppressed only 40-fold, while deletion of NF-κB (−84/+66 deletion mutant) strongly inhibited promoter activity by 1000-fold. Deletion of partial NF-κB (−94/+66) suppressed LTR activity 6-fold (column 1). In the presence of DING p38SJ, there was about a 300- and 200-fold inhibition in each respective case that contained NF-κB binding site (column 3, raw 1-3). For LTR mutants that partially lack NF-κB site (−94/+66), or completely lack NF-κB site (−82/+66), the inhibition by DING p38SJ was about 20- and 6-fold in each respective case (column 3, raw 4-5). When both RPII and TAR regions were deleted, but NF-κB was present (−117/+3), there was about 5-fold LTR inhibition by DING p38SJ (column 2, raw 6). We conclude that DING p38SJ inhibits the transcription activity of the the HIV-1 LTR containing binding sites for cellular transcriptional activators p65 NF-κB and RPII, and viral HIV-1 Tat binding site (TAR). Thus, DING p38SJ has a strong inhibitory effect on HIV-1 LTR transcription.

Our results show that DING inhibits LTR transcription through NF-κB, RPII binding sites, and TAR region observed in in promoter reporter assays (Figure 6A).

### N-terminal region within DING p38SJ that contains phosphate-binding domain is important for the inhibition of p65-associated LTR transcriptional activation

Effect of DING p38SJ and its deletion mutants on NF-κB and LTR activation was studied in promoter/reporter assays in cells expressing p38SJ deletion mutants (Figure 6B). Schematic representation of full-length DING p38SJ and DING deletion mutants are shown in figure 6B, (top panel). Numbers (fold activation) are shown in figure 6B. Results from promoter-reporter assay using DING deletion mutants demonstrate that N-terminal region of DING p38SJ that contains phosphate-binding domain is important for the inhibition of p65-associated LTR transcriptional activation.

## Discussion and Conclusion

Inhibition of HIV-1 and p65 NF-κB phosphorylation by DING is an important event in the controlling of HIV-1 infection and HIV-LTR transcriptional activation. DING p27SJ was first cloned in 2006, then full length DING p38SJ was cloned in 2011 from *Hypericum perforatum* (Genbank: AAW57408.1) [1,2]. DING p27SJ protein is a member of DINGG family of proteins with four conserved N-terminal DINGG sequences. DING proteins also contain phosphate-binding domain, and some are related to alkaline phosphatases. DING proteins play very important roles in human diseases, including viral infections [8].

We demonstrated here the suppressive effect of DING p27SJ and DING p38SJ on p65 subunit of NF-κB (both endogenous and exogenously expressed). The mechanism for this appears to be dephosphorylation of the NF-κB, and the involvement of the nuclear-cytoplasmic translocation. Importantly, p65 co-factor, p50 was also dephosphorylated by DING. This result aligned with the previously published data, indicate depletion of the phosphorylated forms of p65- and p50-NF-κB in nuclei of 1G5 cells treated with the exogenous human variant of DING protein [56]. The current study provided additional information indicating that that the mechanism of inactivation of NF-κB complex by DING p27SJ was I-κB independent. Our data showed that drastic changes in NF-κB levels were not affected by I-κBα expression, but by the presence of DING protein. Interestingly, we found I-κBα also in the nucleus, and this result is aligned with the previously published data by Lain de Lera T, et al., who demonstrated the I-κBα expression in the nucleus of human peripheral blood T lymphocytes [57]. Current models explain that the inhibitory ability of I-κBα is mediated by by retaining Rel/NF-κB proteins in the cytoplasm.

Further, I-κBα was also found in the nucleus, and nuclear fraction of I-κBα was more stable than cytosolic I-κBα [57]. Interestingly, nuclear I-κBα could not inhibit binding of NF-κB to DNA. In addition, it was shown that I-κB was found in the nucleus of resting and PMA-activated human PBL in active form, and was unable to inhibit binding of NF-κB to DNA [57]. Further, it was shown that nuclear presence of I-κBα can lead to the translocation of NF-κB from the nucleus to the cytoplasm [55]. I-κBα, by positioning in the cytoplasm in an inactive form, can regulate the transcriptional activity of NF-κB, but if expressed in the nucleus, it can disrupt NF-κB/DNA binding and even translocation of NF-κB back to the cytoplasm. Thus, suppression of NF-kappa B/DNA binding and the nuclear export of I-κBα is one of essential mechanisms to control NF-κB-dependent gene expression.

One of possible mechanism involved in the inhibition of p65 subunit of NF-κB can be mediated by DING interference with NF-κB nuclear trafficking, as more p65 and p50 NF-κB forms were accumulated in the cytoplasm in DING-expressing cells, while I-κBα was found in the nucleus. Furthermore, DING protein affected PMA-induced activation of NF-κB p65 and its translocation into the nucleus. Again, more cytoplasmic accumulation was observed for p65 in DING-expressing cells. We suggest that the DING-mediated disturbance of the p65/p50 NF-κB nuclear translocation can explain the phenomenon of the inhibition of HIV-1 LTR activation, as there is an insufficient amount of this protein in the nucleus to initiate the HIV-LTR gene transcription. Interestingly, results from the elongation assay demonstrate that the longer is the transcript and the further is a primer from transcription initiation start, the stronger is the inhibitory effect of DING on the elongation of p65 transcript. We established, that DING requires both, NF-κB and RPII binding domains for LTR suppression. Interestingly, the N-terminal region of DING that contains a phosphate-binding domain is important for the inhibition of p65-associated LTR transcriptional activation. In conclusion, DING protein exhibits a phosphatase activity and inhibits phosphorylation of p65 NF-κB, its nuclear translocation and activation, and strongly inhibits HIV-1 replication. The recently published data indicate that NF-κB factor plays an important role in the pathogenesis of other viruses, such as SARS-COV-2, MERS and SARS-COV-1 [58,59]. Thus, the use of DING proteins as therapeutics can be a promising tool in the inhibition of not only the HIV-1, but also other viruses, such as SARS-COV-2. In the absence of current specific treatments for COVID-19, the DING based therapeutic strategies can be developed to treat these diseases as an alternative to the vaccine prevention to end the epidemic.
